# Association between outdoor temperature and achilles tendon repair: A 14-years nationwide population-based cohort study

**DOI:** 10.1371/journal.pone.0265041

**Published:** 2022-03-18

**Authors:** Kwang Hwan Park, Jae Han Park, Yeo Kwon Yoon, Jai Bum Kwon, Jung Hwan Kim, Eunju Lee, Yunho Roh, Seung Hwan Han, Jin Woo Lee

**Affiliations:** 1 Department of Orthopaedic Surgery, Yonsei University College of Medicine, Seoul, South Korea; 2 Department of Orthopaedic Surgery, Catholic University of Daegu School of Medicine, Daegu, South Korea; 3 Biostatistics Collaboration Unit, Department of Biomedical Systems Informatics, Yonsei University College of Medicine, Seoul, South Korea; Istanbul University Istanbul Faculty of Medicine: Istanbul Universitesi Istanbul Tip Fakultesi, TURKEY

## Abstract

The incidence of achilles tendon rupture varies by gender, age, and seasonal variation. However, there has been no study as yet linking achilles tendon rupture to daily fluctuations in outdoor temperature. The purpose of this study was to investigate the association between outdoor temperature and achilles tendon rupture using a Korea Meteorological Administration database and a Korean National Health Insurance Service-National Sample Cohort database. Between 2002 and 2015, all instances of achilles tendon repair were retrieved from the National Health Insurance Service-National Sample Cohort database to examine sociodemographic factors, specifically sex, age, residential area, and income level. Minimum age requirement was 20 years. Outdoor temperatures recorded at 16 observation points in South Korea were also acquired from the Korea Meteorological Administration data center for analysis. Overall, 850 (0.119%) of 713,456 individuals in the National Health Insurance Service-National Sample Cohort database underwent achilles tendon repair between 2002 and 2015. Yearly procedural totals increased with advancing age, peaking at ages 30–39 years (14.6 per 100,000 persons) and declining thereafter. Minimum, median, and maximum outdoor temperatures were associated with achilles tendon repair (*p*<0.05), as did household income. In multivariate logistic regression analysis, outdoor temperatures, sex, age, and household income emerged as factors significantly associated with achilles tendon repair. Outcomes of this study confirm an association between incidence of achilles tendon repair and outdoor temperature, the latter denoting a novel index and likely surrogate measure of vigorous physical activity afforded by warmer weather.

## Introduction

The incidence of achilles tendon rupture is increasing worldwide [1]. In the Danish population, the frequency has increased from 26.95/100,000/year in 1994 to 31.17/100,000/year in 2013 [2]. Lemme et al. have also indicated a significant rise in incidence of achilles tendon rupture within the US populace [3]. It is apparent that achilles tendon rupture totals are climbing in tandem with the number of recreational sports enthusiasts [3–5]. Several recently conducted studies have identified correlations between seasonal change and incidence of achilles tendon rupture/repair [4–6], relying on four-season weather approximations rather than precise outdoor temperatures. Consequently, the applicability to other countries with dissimilar climates is limited.

In this situation with persistent increasing tendency of Achilles tendon rupture, it is important to raise awareness of relevant risk factors to prevent the injury successfully. There are several known risk factors in numerous number of reports, but association between Achilles tendon rupture and outdoor temperature or seasonal variation is not fully understood yet. Some reports demonstrated the relationship between sports injuries and seasonal distribution. The high incidence of ACL injuries in professional soccer players during the summer season and peak incidence of hamstring injuries in football players at the beginning of the season [7,8].

In this study, we hypothesized that the outdoor temperature and achilles tendon repair (ATR) incidence were associated. Instead of invoking vague seasonal categorization, the present study was conducted with the aim of providing more practical information with precise temperatures at the time of injury. Outdoor temperature has greater reasonable relevance in terms of physical activity level like sports or recreational activity [9–14], and they were reported to increase the risk of ATR [3]. So we focused on its association with incidence of ATR. Sociodemographic factors, such as sex, age, household income, and latitudes of residential areas, were likewise culled from the National Health Insurance Service-National Sample Cohort (NHIS-NSC) for analysis. Therefore, the aims of this study were to review the associating factors with ATR and investigate the association between outdoor temperature and ATR. This study can provide useful knowledge regarding the association between ATR and outdoor temperature.

## Materials and methods

### Healthcare database

This retrospective cohort study was conducted in South Korea, where a single national insurance provider (Korean National Health Insurance Service) covers >97% of the population. The data collected originated from a representative sampling (approximately 1,000,000 Koreans) of the National Health Insurance Service-National Sample Cohort (NHIS-NSC, 2002–2015 sample cohort v2.0 database) provided by the Korean National Health Insurance Service. This database archives healthcare eligibility profiles, including sex, age, socioeconomic variables, residential area, eligibility type, household income, and medical treatment data. Diagnostic codes (Korean Classification of Disease, KCD) and codes for clinical procedures were acquired from bills claimed by medical service providers. This study was approved by an institutional review board.

### Meteorological parameters

Daily temperatures and other information recorded at observation areas were provided by the Korea Meteorological Administration (KMA) data center. Data from 16 representative observation stations at latitudes between 33° 14’ and 38° 15’ within South Korea were merged with NHIS-NSC data using the R statistical program. Minimum, median, and maximum temperatures on days of surgery were calculated accordingly. Daily temperature range was calculated as maximum minus minimum temperature recordings each day. Temperatures readings of minimum, median, maximum, and range recorded at times of Achilles tendon repair procedures served to assess the association between ATR and outdoor temperature.

### Sociodemographic factors

Sociodemographic factors archived in the NHIS-NSC database, including sex, age, residential area, and household income, were updated annually. Residential areas were classified as high- or low-latitude regions, reflecting KMA designations. Household incomes were classified by percentile. Samples under the age of 20 were excluded in this study.

### Definition of control group

Control group included individuals who were diagnosed with any disease, except achilles tendon rupture (KCD code S86.0). Because ATR was rare with an occurrence of 0.119% in this study, it was necessary to adjust the ratio between ATR group and control group. We selected the control group by random sampling of the individuals who have not experienced ATR. To optimize a statistical power, the sampling ratio of ATR group and control group were set to 1:4 [15]. In addition, because ATR was increasing year by year, we extracted a control proportional to each year. According to the temperature levels in the control group, the temperatures at the time of reporting to NHIS-NSC database for control individuals were provided by KMA data center.

### Definition of Achilles tendon repair (ATR) group

ATR group included all individuals who were diagnosed with achilles tendon rupture (KCD code S86.0) and were received a surgical repair (billing code N0920, charged on the same day as the date of surgery). For the ATR group, outdoor temperatures of each subject were connected at the time of ATR. Only primary injuries were included in this study, repeat procedures considered grounds for exclusion.

### Annual incidence of achilles tendon repair

During the 14-years study interval (2002–2015), only 46 (0.006%) of 788,399 individuals experienced ATR on more than one occasion. Hence, it was assumed that this study included all ATR events, without a window period. Annual incidence was calculated as of January 1 each year. Follow-up began on January 1 and ended on December 31 each year. The incidence rate was calculated as total ATR events divided by person-years of risk (PYRS) [2]. PYRS was defined as the sum of years each person is at risk of experiencing ATR within a given year. Any person who did not die within the year contributed one year. Because exact dates were retrievable from NHIS-NSC death tables, a death at some point during a year affected the survival period in that year. PYRS was calculated as the number of persons alive during the year minus the survival-compensated number of persons who died during the year. Incidence was then multiplied by 100,000, generating incidence per 100,000 person-years.

### Statistical analysis

Reporting of continuous variables depended on whether or not the assumption of normality was met. If not, data were reported as median (Q1, Q3) and analyzed via Mann-Whitney U test. Categorical variables were presented as frequencies (percentages), using chi-square test for comparisons. Correlations between categorical variables and ATR occurrences were conducted using Fisher`s exact test. A logistic regression model was developed to evaluate independent risk factors associated with ATR. Statistical significance was set at *p*<0.05. All computations relied on standard software (SAS v9.4 [SAS Institute, Cary, NC, USA] and R v3.4.3 freeware [http://www.R-project.org; The R Foundation for Statistical Computing, Vienna, Austria]).

## Results

### Incidence of ATR

**Fig 1:**
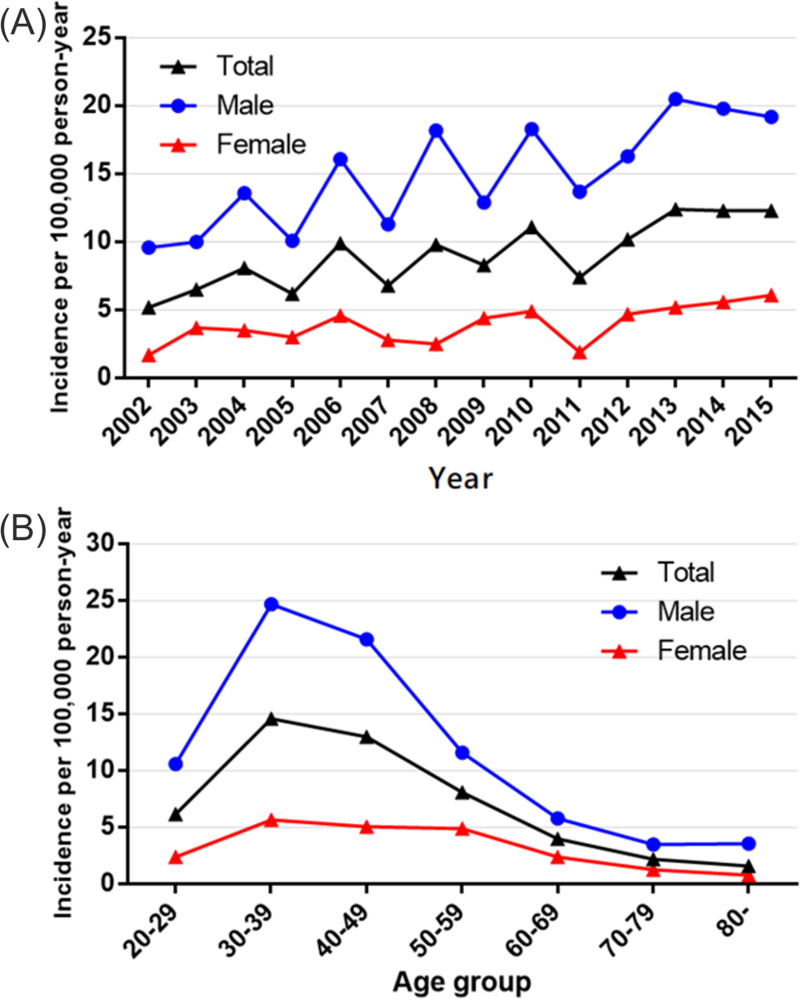
Incidence of achilles tendon repair (ATR) per 100,000 person-years plotted annually (2002–2015) and by age group. (A) incidence of ATR increased yearly, men surpassing women during the study period. (B) incidence of ATR peaked at 30–39 years, gradually declining thereafter.

Overall, 850 (0.119%) of 713,456 registrants in the NHIS-NSC database underwent ATR between 2002 and 2015. Table 1 shows the annual incidence of ATR for this time interval. There were 850 cases of ATR (men, 653; women, 197) per 9,212,320 person-years. The incidence was 9.2 cases per 100,000 person-years (men, 15.4; women, 4.0). The overall male-to-female ratio was 3.9. Annual ATR incidence rates ranged from 5.2–12.4 per 100,000 person-years during the study period, with a gradual increase from 5.2 in 2002 to 12.4 in 2013 and subsequent decline to 12.3 in 2014 and 2015 (Table 1 and Fig 1).

**Table 1:** Incidence of achilles tendon repair per 100,000 person-years, listed annually.

	Men + Women			Men			Women			Male-to-female ratio
Year	Person-years	No.	Incidence	Person-years	No.	Incidence	Person-years	No.	Incidence	
2002	520,050	27	5.2	229,032	22	9.6	291,018	5	1.7	5.6
2003	540,742	35	6.5	239,801	24	10.0	300,941	11	3.7	2.7
2004	570,218	46	8.1	257,238	35	13.6	312,980	11	3.5	3.9
2005	611,441	38	6.2	278,186	28	10.1	333,255	10	3.0	3.4
2006	637,080	63	9.9	292,335	47	16.1	344,745	16	4.6	3.5
2007	651,807	44	6.8	300,166	34	11.3	351,641	10	2.8	4.0
2008	664,562	65	9.8	307,195	56	18.2	357,367	9	2.5	7.2
2009	683,236	57	8.3	317,597	41	12.9	365,639	16	4.4	3.0
2010	693,610	77	11.1	323,005	59	18.3	370,605	18	4.9	3.8
2011	704,502	52	7.4	328,711	45	13.7	375,791	7	1.9	7.3
2012	718,648	73	10.2	336,763	55	16.3	381,885	18	4.7	3.5
2013	727,353	90	12.4	341,641	70	20.5	385,712	20	5.2	4.0
2014	740,759	91	12.3	349,085	69	19.8	391,674	22	5.6	3.5
2015	748,312	92	12.3	353,247	68	19.2	395,065	24	6.1	3.2
**Total**	**9,212,320**	**850**	**9.2**	**4,254,002**	**653**	**15.4**	**4,958,318**	**197**	**4.0**	**3.9**

No., Number of ATR.

Table 2 shows the incidence of ATR according to age group. The incidence peaked at ages 30–39 years (14.6 per 100,000 person-years), both in men (24.7 per 100,000 person-years) and in women (5.7 per 100,000 person-years). Thereafter, ATR incidence rates gradually dwindled to 1.6 overall in the age group >80 years. The male-to-female ratio at ages 30–39 years was 4.4 (Table 2).

**Table 2:** Incidence of achilles tendon repair per 100,000 person-years, grouped by age.

	Men + Women			Men			Women			Male-to-female ratio
Age group	Person-years	No.	Incidence	Person-years	No.	Incidence	Person-years	No.	Incidence	
20–29	1,621,246	100	6.2	744,033	79	10.6	877,213	21	2.4	4.4
30–39	1,995,308	291	14.6	933,571	231	24.7	1,061,737	60	5.7	4.4
40–49	2,045,594	266	13.0	979,545	212	21.6	1,066,049	54	5.1	4.3
50–59	1,640,504	133	0.8	784,364	91	11.6	856,140	42	4.9	2.4
60–69	1,079,408	43	4.0	498,973	29	5.8	580,435	14	2.4	2.4
70–79	646,809	14	2.2	257,650	9	3.5	389,159	5	1.3	2.7
≥80	183,451	3	1.6	55,866	2	3.6	127,585	1	0.8	4.6

No., Number of ATR.

### Association between ATR and patient variables, specifically outdoor temperature

**Fig 2:**
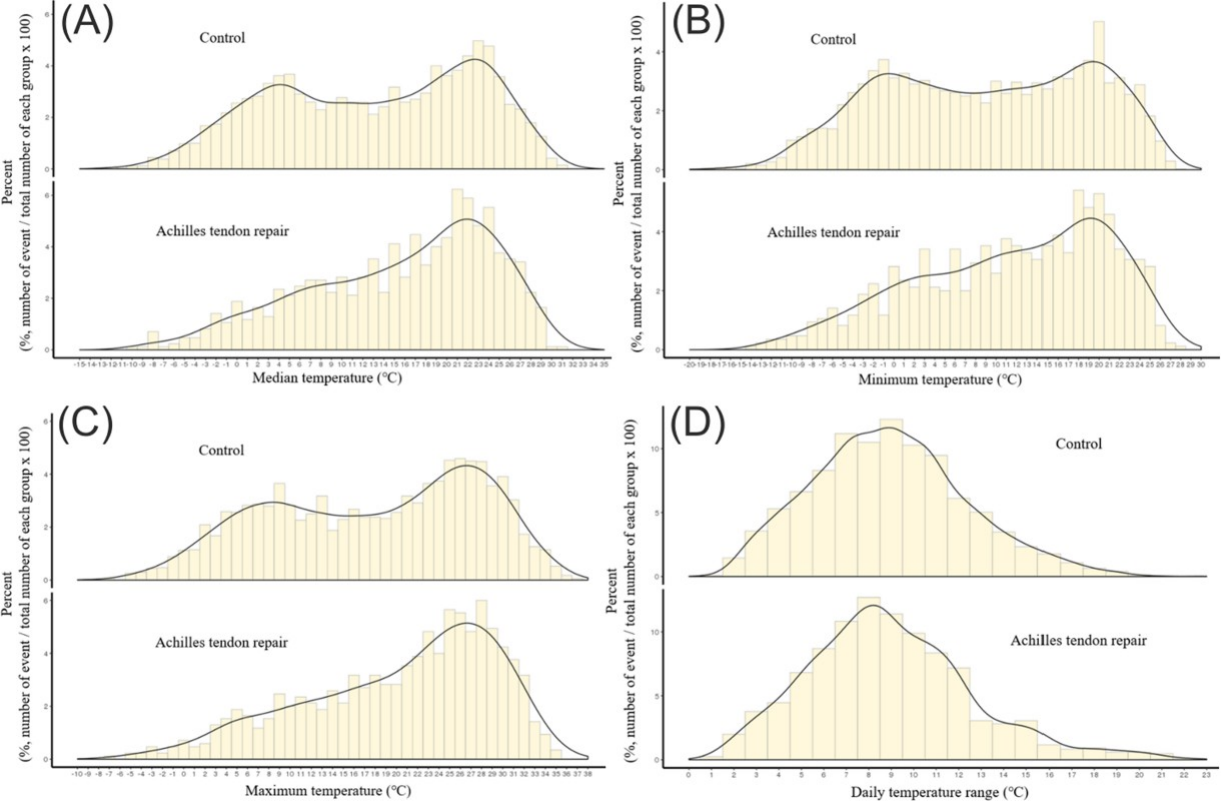
Distribution of temperature index in control group and achilles tendon repair group (2002–2015). (A-C) significant upward shifts in distributions of (A) median, (B) minimum, and (C) maximum temperatures of ATR group, compared with controls; (D) no significant difference in daily temperature ranges of ATR and control groups.

**Fig 3:**
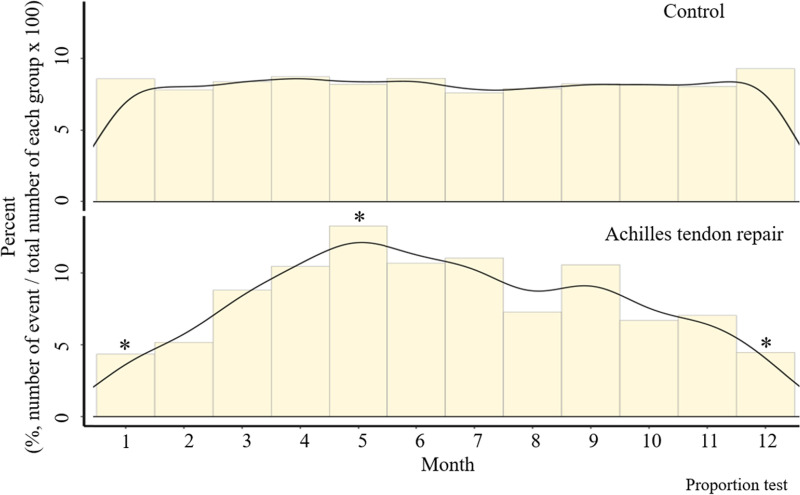
Monthly distribution of occurrences in control and Achilles tendon repair (ATR) groups (2002–2015). Incidence of ATR peaks in May, fewer injuries occurring in December and January.

In comparative analysis of ATR group and control group randomly selected from the NHIS-NSC database, higher outdoor temperatures were associated with increased risk of ATR (Table 3). Minimum, median, and maximum temperatures (°C) in the ATR group exceeded those of the control group (17.4 vs 14.0, 12.7 vs 9.5, and 22.5 vs 19.3, respectively). Distributions of minimum, median, and maximum temperatures in the ATR group shifted significantly higher, compared with the control group (Fig 2). However, daily temperature ranges were not significantly different in the two groups. In addition, there was no significant group difference in residential latitude and higher household income was also associated with increased ATR (Table 3). When stratifying occurrences monthly in the two groups (Fig 3), ATR showed significant peaks in May (*p*<0.05), with significantly fewer injuries sustained in December and January (*p*<0.05 each).

**Table 3:** Comparative analysis of outdoor temperatures in achilles tendon repair group and randomly selected controls (2002–2015).

	ATR (n = 850)	Control (n = 3400)	*p* value
Median temperature, °C	17.4 (9.1–22.6)**	14 (4.6–21.8)**	**< .05**
Quantile of median temperature, °C			**< .05**
Q1 (≤5.3)	134 (15.76)*	933 (27.44)*	
Q2 (>5.3, ≤14.9)	214 (25.18)*	844 (24.82)*	
Q3 (>14.9, ≤22.0)	263 (30.94)*	805 (23.68)*	
Q4 (>22.0)	239 (28.12)*	818 (24.06)*	
Minimum temperature, °C	12.7 (4.3–19.2)**	9.5 (0.4–18.1)**	**< .05**
Maximum temperature, °C	22.5 (14.2–27.5)**	19.3 (9.4–26.4)**	**< .05**
Daily temperature range, °C	8.6 (6.5–11.1)**	8.8 (6.5–11)**	0.576
Latitude of residence area, n			0.890
High latitude (37–38)	410 (48.24)*	1649 (48.5)*	
Low latitude (33–36)	440 (51.76)*	1751 (51.5)*	
Household income, percentile			**< .05**
Low (<30)	126 (15.38)*	710 (22.44)*	
Intermediate (30–70)	285 (34.80)*	1202 (37.99)*	
High (≥70)	408 (49.82)*	1252 (39.57)*	

ATR, achilles tendon repair. Control group members randomly selected from National Health Insurance Service-National Sample Cohort, proportional to yearly ATR group total. Data expressed as numerical values (%)* or median temperature (Q1,Q3)** unless otherwise specified, boldface indicating statistical significance (*p* < .05).

### Analysis of ATR-related risk factors

Results of univariable logistic analysis, exploring associations between meteorological or sociodemographic factors and ATR, are presented in Table 4. Factors displaying significant associations with ATR included median temperature, quantile of median temperature, sex, age, and income. In multivariable logistic analysis excluding median temperature quantile, high median temperature (OR = 1.03), male sex (OR = 4.26), age (OR = 0.95), and higher income level (OR = 1.99) showed significant associations with increased risk of ATR. In multivariable logistic analysis excluding median temperature, higher quantile of median temperature (Q2: OR = 1.70; Q3: OR = 2.35; Q4: OR = 2.14), male sex (OR = 4.24), young age (OR = 0.95), and higher income level (OR = 1.97) were significantly associated with increased ATR risk.

**Table 4:** Logistic regression analyses of factors associated with achilles tendon repair.

	Univariable		Multivariable 1		Multivariable 2	
	*Odds ratio (95% CI)*	*p* value	*Odds ratio (95% CI)*	*p* value	*Odds ratio (95% CI)*	*p* value
Median temperature, °C	1.03 (1.02–1.04)	< .05	-	-	1.03 (1.02–1.04)	**< .05**
Quantile of median temperature, °C		< .05			-	-
Q1 (≤5.3)	ref		ref		-	-
Q2 (>5.3, ≤14.85)	1.77 (1.4–2.23)	< .05	1.70 (1.31–2.21)	**< .05**	-	-
Q3 (>14.85, ≤22)	2.28 (1.81–2.86)	< .05	2.35 (1.82–3.03)	**< .05**	-	-
Q4 (>22)	2.03 (1.61–2.56)	< .05	2.14 (1.65–2.77)	**< .05**	-	-
Male sex	4.41 (3.71–5.24)	< .05	4.24 (3.52–5.11)	**< .05**	4.26 (3.54–5.14)	**< .05**
Age	0.95 (0.94–0.96)	< .05	0.95 (0.94–0.95)	**< .05**	0.95 (0.94–0.95)	**< .05**
Income level (percentile)		< .05				
Low (<30)	ref		ref		ref	
Intermediate (30–70)	1.34 (1.06–1.68)	**0.013**	1.21 (0.94–1.55)	0.135	1.22 (0.95–1.57)	0.112
High (≥70)	1.84 (1.47–2.29)	< .05	1.97 (1.54–2.51)	**< .05**	1.99 (1.56–2.54)	**< .05**
Regional latitude						
High (37–38 degrees)	ref		-	-	-	-
Low (33–36 degrees)	1.01 (0.87–1.18)	0.890	-	-	-	-

Boldface indicates statistical significance (*p* < .05), CI, confidence interval.

## Discussion

The most important finding of the present study was that outdoor temperature was associated with ATR in a 14-years nationwide population-based cohort. Ultimately, we observed a significant association between higher median temperature and increased risk of ATR (OR = 1.03) and the incidence rates ranged from 5.2–12.4 per 100,000 person-years.

Similar to our results, acute rupture of the achilles tendon is one of the most common tendon injuries in adult populations, with incidences of 1.8–59.5 per 100,000 person-years [3,16–19]. In the US, one particular source has analyzed data from the National Electronic Injury Surveillance System (NEISS), demonstrating an increase from 1.8 per 100,000 person-years in 2012 to 2.5 per 100,000 person-years in 2016 and an overall incidence of 2.1 per 100,000 person-years [3]. Younger men (20–39 years) were at greatest risk for such injury. Another study of US military cadets revealed 29 achilles tendon repairs among 93,224 recruits during a 2-year period [20]; and there were 865 achilles tendon repairs among 4,847,093 US military members in a separate 3-year analysis [17]. These researchers found a significantly greater risk of achilles tendon repairs in black (vs nonblack) patients. In Canada (Edmonton), the incidence of achilles tendon rupture between 1998 and 2002 reportedly ranged from 5.5–9.9 ruptures per 100,000 inhabitants, with an overall median of 8.3 ruptures per 100,000 [21]. Occurrences were most frequent in 30–39 and 40–49 age groups of men and women, respectively.

A number of studies investigating the incidence of ATR in Europe and Oceania have been published as well. In Scotland, the overall incidence of ATR increased from 4.7 per 100,000 in 1981 to 6.0 per 100,000 in 1994 [22]. Furthermore, a bimodal distribution was observed in terms of age at time of occurrence, peaking at 30–39 years and at >80 years. In our cohort, we identified a single peak only at 30–39 years, with ATR steadily declining thereafter. Annual incidences of achilles tendon rupture in Denmark have increased from 18.2 per 100,000 inhabitants in 1984 to 37.3 per 100,000 in 1996 [23]. The peak incidence of sport-related ruptures occurred at ages 30–49 years. The average incidence of achilles tendon rupture (per 100,000 person-years) in Finland has similarly risen from 2.1 in 1979 to 21.5 in 2011; and in the UK, the average incidence reported between 1996 and 2000 was 11.3 [16,24]. Data from the Swedish Hospital Discharge Register (SHDR) collected between 2001 and 2012 shows the incidence of achilles tendon rupture increasing from 47.0 to 55.2 per 100,000 person-years in men and from 12.0 to 14.7 per 100,000 person-years in women [19]. In New Zealand (southern hemisphere), the approximate incidence of achilles tendon rupture has been reported as 24.0 per 100,000 person-years during an 8.5-year study [25].

The incidence of ATR determined in this study (approximately 9.6 per 100,000 person-years) was comparable to figures reported globally. Although not immediately clear, we suspect that the recent upsurge was fueled by a growing number of young adults who participate in high-demand sports.

To our knowledge, few studies have focused on seasonal variations in ATR, which are still contested in the literature [4–6]. Findings of a Canadian (Vancouver) study support a distinct seasonal pattern [5]. Among patients with ATR, the highest case numbers accrued during spring (all sports-related) and the lowest during fall and winter. Unfortunately, incidence rates were not provided, given the retrospective, single-center nature of this review. Caldwell et al. also documented significantly more ruptures in spring than in fall [4] when analyzing claims data of a major academic orthopedic surgery department in New York City. Their results suggest that the incidences of injuries related or unrelated to sports follow similar trends. However, Ann et al. of Denmark showed the highest incidence of ATR during fall (peaking in September) and the lowest incidence during summer [2], likely coinciding with the launch of all major sports activities after summer holidays. Also, Saarensilta et al. reported ATR incidence was significantly highest during winter and spring, and lowest during summer in Sweden [6]. Nevertheless, certain past studies have failed to show significant seasonal differences in ATR. Maffulli et al. retrospectively reviewed 15 years of records collected by the National Health Service in Scotland, finding a declining trend in early fall but no substantial seasonal fluctuations in incidence of ATR [22]. In Canada (Edmonton), Suchak et al. performed a retrospective chart review of patients with achilles tendon rupture, demonstrating no significant seasonal differences in incidence [21]. Such mixed outcomes make it difficult to reach a consensus on this issue. Extrapolation to other countries and regions of the world is tenuous, knowing how climates (especially daily outdoor temperatures) may vary.

As in past reports from Vancouver and New York City, we recorded the highest and lowest incidences of achilles tendon repair/rupture in spring and winter. Weather in these regions is similar, the temperatures averaging 10.4°C, 12.8°C, and 12.5°C annually in Vancouver, New York City and Seoul (capital of South Korea), respectively. It is thus reasonable to conclude that seasonal variations in ATR may be shared by regions with similar outdoor temperatures that can eventually affect the participation of sport activity. The current analysis subsequently relied on precise daily temperatures rather than equivocal seasonal categorizations. Daily minimum, median, and maximum temperatures ultimately proved significantly higher in the ATR group, compared with control subjects. Outdoor temperature, unless it is too high, may then be a valid index for predicting risk of ATR. This was not true of regional latitude, which had no significant impact on incidence of ATR in our study. The minor latitude differences (33–38 degrees) were ostensibly culpable, imparting no significant differences in temperatures among regions. Further studies are needed to validate temperature as an index of ATR in a country with major latitude departures.

Achilles tendon rupture is generally linked to a number of risk factors, including male sex, young age, body mass index, race, smoking status, fluoroquinolone or corticosteroid use, history of prior Achilles tendinopathy, blood type O, diabetes and other medical comorbidities, and participation in sports activities [17,21,25–28]. Owing to constraints of claims data, only some of these known risk factors were analyzed, focusing on sex, age, household income, regional latitude, and temperature index. Our logistic regression model identified male sex, younger age, higher income, and median temperature as significant variables associated with ATR, generating odds ratios of 4.26, 0.95, 1.99, and 1.03, respectively. After adjustment for confounding factors, median temperature displayed a significant association with ATR and may serve as a novel risk factor within the region of South Korea.

Clear mechanisms underlying the relationship between outdoor temperature and ATR are not fully elucidated. We supposed that high outdoor temperature is able to exert an influence on ATR in several ways. First, it induces changes in behavior to perform more activity in outside including sports [29]. Various researchers have noted a positive association between outdoor temperature and level of physical activity [30–34]. Physical activity is clearly seasonal, intensifying in spring and summer [9–14], and periods of peak household or leisure-time physical activity are known to overlap with periods of warmest temperatures [35,36]. There is also prior documentation of reduced physical activity during the winter season [9], and as shown by a study done in Galveston, TX (average July temperature, 29°C), extreme heat is another recognized deterrent [37]. In other previous reports, high temperature increased the risk of injuries by sport, recreational activity and occupational injury [29,38]. These publications provide some rationale for the observed peaking of ATR incidence in May (average temperature, 17.8°C). Second, it may have direct effect on trauma itself, by inducing muscle fatigue and dehydration [39]. In the environment with high temperature, dehydration influences muscular endurance and muscular strength [38,40].

The strength of this study was our access to nationwide, population-based data, reflecting the true incidence of ATR in South Korea and outcomes relevant to all its inhabitants. A large cohort of South Korean adults, each reliably diagnosed as ATR and properly covered, comprised the study population. Hence, the rising yearly incidence of surgically treated ATR was presumed valid. In addition, use of claims data from a National Health Insurance repository was highly advantageous in curbing the expense of a population-based study. It allowed us to identify patients with ATR and calculate the incidence throughout South Korea in a cost-effective manner. The precision of such actions may not be ensured, but this strategy worked well for estimating general trends. Additionally, this study may have implications for the planning of injury prevention strategy and reducing substantial injuries under the current worldwide climate change tendency.

We must similarly acknowledge the following limitations imposed by our dependence on claims data: (1) lack of other clinical variables, including cause of injury, side of injured tendon, medical and surgical comorbidities at time of injury, history of medication use, and level of participation in sports; (2) a nonsurgical achilles tendon rupture treatment void; (3) exclusion of patients <20 years old; (4) generalization problems due to race, ethnicity, and geographic restrictions, and (5) a date difference between the rupture and the surgery. Despite our discounting of nonoperative achilles tendon rupture treatment, we have shown sufficient evidence of an association between ATR and temperature. Also, achilles tendon rupture seldom occurs in the youngest population subset (<20 years) [23,25,41], so our estimates of ATR incidence seem adequate. To generalize the impact of temperature on ATR, analogous studies conducted within other countries are warranted.

## Conclusions

This effort is the first to verify relatively high outdoor temperature as a novel risk factor for achilles tendon rupture. The more vigorous physical activity prompted by warmer weather may offer a plausible explanation. Awareness of risk factors and proper education should help prevent achilles tendon rupture on a broader basis.
